# DALK combined intralamellar tectonic patch graft: an alternative approach to treat frank corneal perforation

**DOI:** 10.1186/s12886-023-03179-7

**Published:** 2023-10-27

**Authors:** Gege Xiao, Hanzhi Ben, Shaofeng Gu, Jing Hong

**Affiliations:** Department of Ophthalmology, Peking University Third Hospital, Beijing, China

**Keywords:** Corneal perforation, Deep anterior lamellar keratoplasty, Intralamellar patch graft, Autologous graft

## Abstract

**Background:**

Deep anterior lamellar keratoplasty (DALK) has gained popularity in cases of corneal thinning and leaking descemetocele. In this study, we introduced an intralamellar tectonic patch graft in addition to conventional DALK procedures to treat frank cornea perforation.

**Methods:**

This retrospective case series included 13 patients (13 eyes) with frank corneal perforations who underwent DALK combined with intralamellar tectonic patch graft between December 2015 and December 2021. In addition to the standard DALK procedure, the perforation site was repaired with an extra intralamellar tectonic patch graft. The collected data included patient demographics, aetiology, size and location of the corneal perforation, visual acuity, surgical details, and postoperative complications.

**Results:**

Seven patients underwent autologous intralamellar patch grafts, whereas six received allogeneic ones. Anatomical success was achieved in all patients. The mean postoperative follow-up was 33.31 ± 25.96 months (6–73 months). The postoperative visual acuity (0.90 ± 0.65 logMAR) was significantly improved (P = 0.003) compared to the preoperative score (1.74 ± 0.83 logMAR). Best corrected visual acuity (BCVA) improved in 12 eyes (92.3%). The mean endothelial cell density was 2028 ± 463 cells/mm^2^, 6–12 months postoperatively. There was no recurrence of perforation, and the anterior lamellar graft remained transparent in 12 patients (92.3%). Postoperative complications included epithelial defects (23.1%), ocular hypertension (15.4%), and cataract (7.7%).

**Conclusions:**

DALK combined with intralamellar tectonic patch graft may serve as a secure and effective alternative in treating frank corneal perforation, with reduced complications compared to conventional penetrating keratoplasty.

**Supplementary Information:**

The online version contains supplementary material available at 10.1186/s12886-023-03179-7.

## Introduction

Corneal perforation is the breakdown of the outer shell of the eye and it is a medical emergency that requires urgent treatment, as the exposure of the eye content to the external environment may lead to severe ocular morbidities; the worst result could be enucleation [1]. Corneal perforation can be classified as traumatic or non-traumatic, with non-traumatic perforation further divided into infectious or sterile. The aetiology in sterile conditions include corneal degeneration, autoimmune disease, neurotrophic ulcers, and ocular surface diseases, such as keratoconjunctivitis sicca [2–4]. Cyanoacrylate glue, amniotic membrane, and pedicle conjunctival flap have proven to be effective but only as temporary solutions; long-term improvement of visual acuity often demands corneal transplantation [5, 6].

Although tectonic deep anterior lamellar keratoplasty (DALK) has gained popularity over the years, it was primarily performed in descemetocele and impending perforation [7–9]. Penetrating keratoplasty (PK) remains the gold-standard treatment to restore globe integrity, especially in cases of large, frank corneal perforation [6, 7, 10–13]. However, studies have shown a significant incidence of complications like secondary glaucoma, endothelial rejection and subsequent graft failure [7, 11, 14–16]. Emergency PK, a common emergency measure for corneal perforation, was reported to have an even higher rate of graft failure [17]. Furthermore, the shortage of optical quality donor tissues in East Asian countries remains an unavoidable constraint on performing PK. Herein, we introduced an intralamellar patch graft in addition to conventional DALK procedures to treat frank corneal perforation.

## Methods

### Study design

This retrospective study included 13 patients (13 eyes) with frank corneal perforations who underwent DALK combined with intralamellar patch graft between December 2015 and December 2021 at Peking University Third Hospital. All interventions were performed by the same surgeon (J. H.) under local anesthesia. This study was conducted in accordance with the principles outlined in the Declaration of Helsinki.

### Surgical technique

The cornea was fully exposed using a lid speculum. The globe was fixed by fixation sutures through the superior and inferior recti. The prolapsed iris was adequately rinsed with a balanced salt solution and tobramycin. In patients who presented with a crumbly iris, the iris was excised as they have a higher chance of getting infected; otherwise, the iris was preserved as much as possible and returned to the anterior chamber.

Since the size and location of the perforation varied considerably, we adopted different types of DALK from circular, semilunar, to crescentic. The anterior lamellar grafts were harvested from the anterior corneal cap of the pre-cut donor cornea which was prepared for Descemet-stripping automated endothelial keratoplasty (DSAEK). The uniform thickness of the DSAEK graft was 150–200 μm, so there would be nearly 400 μm left for lamellar keratoplasty.

**Fig. 1:**
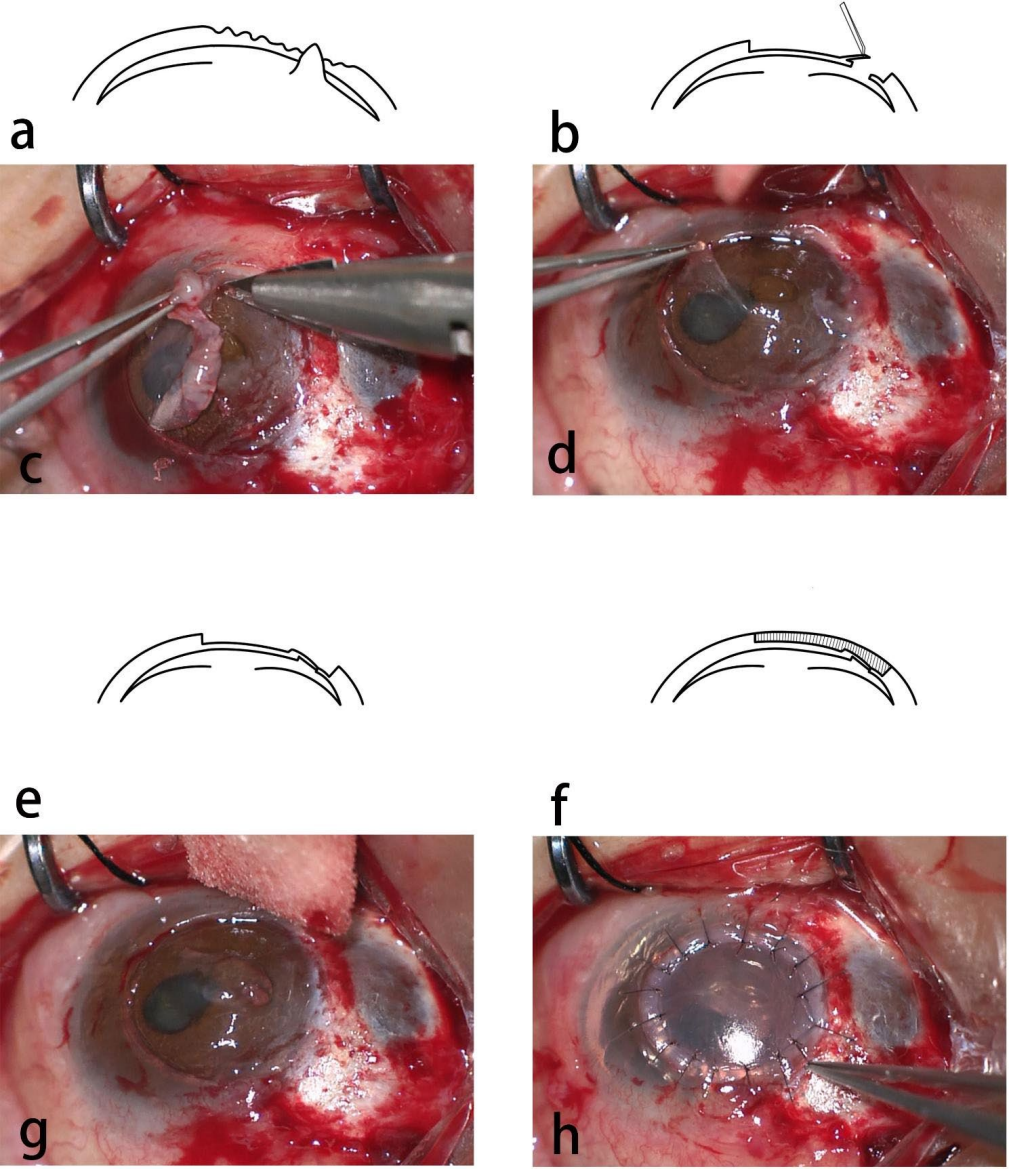
Schematic diagram and photographs taken during the surgical process. **a, c** The necrotic stroma was excised during the preparation of recipient bed. **b, d** The autologous patch was peeled off from the surrounding intact stroma. Noticed the prolapsed iris through the perforation (the white arrow). **e, g** The autologous patch (the grey arrows) was flipped over to cover the perforation site and was secured by interrupted 10/0 nylon sutures. **f, h** The anterior lamellar graft was sutured in place in the end

The recipient bed was prepared by manually dissecting the stroma layer by layer. After the complete excision of the pathological stroma, if the deep stroma remained intact, we would further peel off a piece of stroma layer approximately 20 μm, namely the autologous intralamellar patch graft, at the top of the remaining stroma and flip it over to cover the perforation site (Fig. 1). Otherwise, an allogeneic intralamellar patch graft had to be used to cover the sunken perforation area. The allogeneic intralamellar patch graft was harvested from the margin of the anterior corneal cap of pre-cut donor cornea prepared for DSAEK. The allogeneic patch graft (including the endothelium, the Descemet membrane and the posterior stroma adherent to the Descemet membrane) was secured to the perforation site by 2 to 3 interrupted 10/0 nylon sutures. If the perforation were larger than 3 mm, the autologous patch graft would need to be sutured too. After the perforation site was blocked, the anterior DALK graft was transferred onto the recipient bed and sutured in place by 16–20 interrupted 10/0 nylon sutures (Supplemental video 1).

In the severe iris synechia condition, viscoelastic material was used to separate the iris from the posterior cornea. An air bubble was injected to reformed the anterior chamber and the pupil gradually returned to its normal shape. Finally, a bandage contact lens was used to protect the postoperative ocular surface and to promote epithelial healing.

In one case, severe necrosis of the nasal peribulbar conjunctiva led to a conjunctival autograft. In four cases of autoimmune disease and one case of neurotrophic ulcer, an amniotic membrane patch graft was applied, while the latter also underwent tarsorrhaphy. Postoperative treatment included antibiotics to prevent infection, steroids to ease inflammation, topical immunosuppressive agents to restrain graft rejection, and artificial tears to promote epithelial healing.

### Outcome measures

Anatomical success was defined as achieving tectonic integrity once and for all so that no further intervention out of tectonic purpose should be required. In determining the postoperative graft-hose interface haze, the grading system was as follows: mild for minimal interface haze where iris details are blurry but still visible; moderate for cases where only pupil edges can be seen; severe for cases where the pupil edges are not visible. These eyes were evaluated and graded by the same ophthalmologist performing the surgery who was fully aware of the nature of the intralamellar patch graft. Anterior segment optical coherence tomography (ASOCT) was used to look for interface fluid and observe the gradual assimilation of the intralamellar patch graft. Confocal microscopy was used to measure the endothelial cells density.

### Statistical analyses

Statistical analyses were performed using SPSS 22.0. Changes in Best corrected visual acuity (BCVA) was analyzed using the Wilcoxon signed-rank test. Statistical significance was set at *P* < 0.05.

## Result

The study included 13 eyes of 13 patients (7 males and 6 females) with a mean age of 52.38 ± 18.51 years old (ranging from 16 to 71). The aetiology included microbial keratitis (4, 30.8%), autoimmune disease (3, 23.1%), blepharokeratoconjunctivitis (3, 23.1%), marginal degeneration (1, 7.8%), neurotrophic keratitis (1, 7.8%), and traumatic injury (1, 7.8%). Details of the individual cases are listed in Table 1.

**Table 1:** Summary of the Individual Cases

Case No.	Age & gender	Etiology of perforation	Time from symptoms onset	Location & size (mm)	Preop VA	Intralamellar graft	Postop BCVA	Postop ECD	Postoperative complications	Comments
1	66 & M	Herpetic keratitis	20 days	Inferior & 2*4.5	HM/20 cm	Allo	0.04	Not recorded	Nil	
2	39 & F	BKC	3 months	Inferonasal & 2*1.5	0.02	Allo	0.3	2568	Nil	
3	64 & M	Marginal degeneration with trauma history	7 days	Superior & 4*1	0.04	Auto	0.08	1935	Nil	
4	53 & F	Fungal keratitis	11 days	Inferior & 4*2 with hypopyon	LP	Allo	0.15	1523	Cataract	ECCE + IOL 15 months later
5	46 & M	Traumatic injury	2 months	Inferior & 3*3	0.08	Auto	0.1	1860	Nil	
6	71 & F	Rheumatic Arthritis	9 days	Inferior, double & 2*2.5 plus 2*2	HM/10 cm	Auto	0.5	1045	Elevated IOP; Epithelial defects	ECCE + IOL 10 months later
7	42 & M	Neurotrophic ulcer	8 days	Inferior & 2*3	0.06	Auto	0.06	Not recorded	Nil	Tarsorrhaphy 2 days later
8	62 & F	Bacterial keratitis secondary to dacryocystitis	7 days	Nasal & 1*3	FC/20 cm	Auto	0.5	2380	Nil	ECCE + IOL without suture removal 2 years later;
9	71 & M	Herpetic keratitis	20 days	Inferior & 5*6	LP	Allo	0.06	Not recorded	PCED	PK + ECCE + IOL half a year later
10	16 & M	BKC	3 months	Inferior & 2*4	0.6	Allo	0.8	2076	Nil	
11	21 & M	BKC	2 days	Inferonasal & 2.5*2	0.4	Allo	0.8	2400	Epithelial defects	
12	60 & F	Ocular cicatricial pemphigoid	1 day	Nasal, central & 2*3	HM/10 cm	Auto	HM/30 cm	2344	Nil	
13	70 & F	Mooren ulcer	1 day	Nasal & 2*1	FC/30 cm	Auto	0.06	2154	Nil	

Abbreviations: No.=Number; M = Male; F = Female; BKC = Blepharokeratoconjunctivitis; BCVA = Best-Corrected Visual Acuity; FC = Finger Counting; HM = Hand Motions; LP = Light Perception; Allo = Allogenic; Auto = Autologous; ECD = Endothelial Cells Density; IOP = Intraocular Pressure; PCED = Persistent Corneal Endothelial Defects; ECCE = Extra-Capsular Cataract Extraction; IOL = Intraocular Lens implantation

Three patients had received initial treatment at their local hospital. Case No.4 had undergone a lamellar keratoplasty but soon suffered from perforation recurrence. Case No.10 had received a conjunctival flap, but the perforation site continued to leak. Case No.12 had undergone an amniotic membrane transplantation. However, the amniotic membrane fell off a week after the surgery and the remaining suture leaded to a severe rejection. The other ten patients presented with untreated, leaking perforation. In case No.8, We performed an allogeneic scleral patch as a temporizing tectonic measure while removing the infectious dacryocyst.

**Fig. 2:**
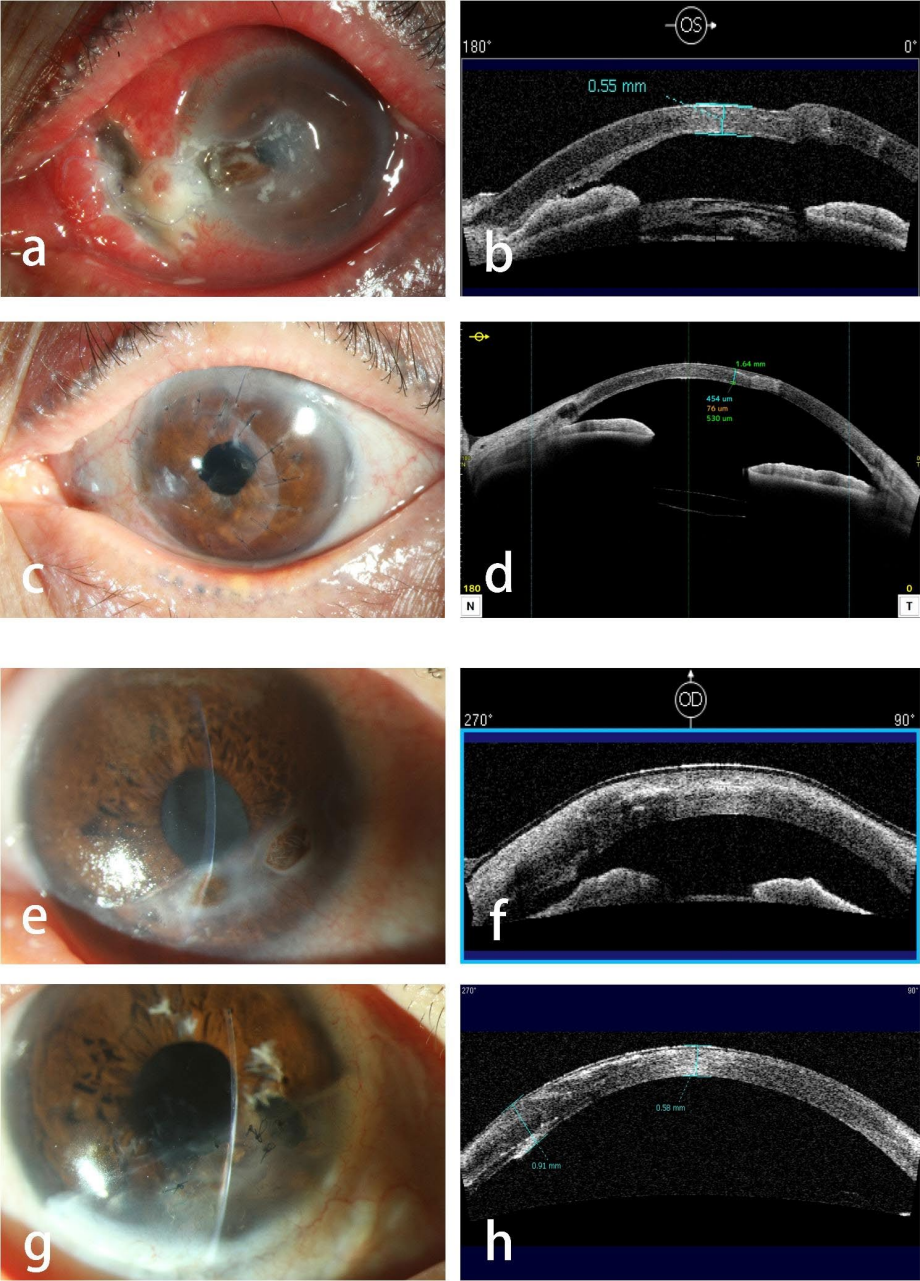
**a** Preoperative photo of a 62-year-old woman with infectious keratitis secondary to dacryocystitis. **b** ASOCT taken by one month after the surgery. **c, d** Photograph and ASOCT taken by 2 years after the surgery. **e** Preoperative photo of a 71-year-old woman with rheumatoid arthritis. **f** ASOCT taken by two weeks after the surgery. **g, h** Photo and AS-OCT taken by 2.5 years after the surgery. Noticed that the repaired perforation area turned flat and the intralamellar patch had been successfully assimilated into the stroma

During the DALK combined with intralamellar patch graft procedure, seven patients underwent autologous intralamellar patch grafts, while six received allogeneic grafts. Anatomical success was achieved in all patients. The mean postoperative follow-up was 33.31 ± 25.96 months (6–73 months). The postoperative visual acuity (0.90 ± 0.65 LogMAR) was significantly improved (P = 0.003) compared to the preoperative visual acuity (1.74 ± 0.83 LogMAR). Postoperative ASOCT showed favorable reconstruction of the anatomical structure (Fig. 2). The mean endothelial cell density was 2028 ± 463 cells/mm^2^, 6–12 months postoperatively. In 12 cases (92.3%), the anterior stromal graft remained transparent. Among them, five intralamellar patch grafts showed mild graft–host interface haze; six showed moderate haze; one showed severe haze. The comparison of the interface haze between autologous and allogeneic intralamellar groups is presented in Table 2.

**Table 2:** Comparison of Interface Haze Between Autologous and Allogenic groups

	autologous	allogenic
Mild	4	1
Moderate	3	3
Severe	0	1
Graft failure	0	1^*^

^*^No.7 patient suffered graft failure due to persistent corneal epithelial defects

No graft rejection or perforation recurrence was observed; however, a few short-term complications have been reported. Four patients underwent cataract extraction and intraocular lens implantation, among which three had already got cataracts before the keratoplasty. Elevated intraocular pressure (IOP) caused by residual viscoelastic material was detected in two eyes. An anterior chamber tap was performed, followed by an intravenous injection of Mannitol 20%, and the IOP dropped to normal within two days. Epithelial defects were detected in three eyes; with the administration of bandage contact lens as well as topical agents including epidermal growth factor and preservative-free artificial tears, two of them were recovered while the other (No.9) developed persistent corneal epithelial defects (PCED). The patient had been diagnosed with herpetic keratitis two years ago and had suffered from recurring virus relapses despite receiving medication treatment at the local hospital. After six months of anti-viral therapy (including topical corticosteroids, topical ganciclovir gel, and oral ganciclovir) and preservative-free artificial tears treatment, the epithelium lesion showed no improvement and we had to perform PK for this patient.

## Discussion

PK is the most common type of tectonic keratoplasty performed for corneal perforation. Although initial anatomical success is usually achieved, graft survival differs due to the immune rejection. Graft clarity rate after the PK ranged from 44.55 to 71.0% at the last follow-up [11, 17–19]. Immune graft rejection, recurrence of primary disease and surface disorders, mostly PCED, were the mean causes of graft failure. Immune graft reaction, identified as progressive endothelial decompensation combined with cell precipitates, was reportedly 16.7–19% in patients who underwent PK [13, 19]. Furthermore, since frank corneal perforation demands immediate treatment, emergency keratoplasty is more frequently performed. Reinhard et al. reported that grafts following emergency PK suffered a higher incidence rate of immune rejection than grafts following scheduled PK (37.2% vs. 21.4%), probably due to the acute inflammation [17]. In our study, only three patients had received temporizing tectonic treatment beforehand, while the other ten patients presented with leaking, untreated corneal perforation, thus requiring urgent intervention. Performing DALK combined with the intralamellar patch graft instead of PK has successfully reduced the incidence of immune rejection. In addition, Roberts et al. have demonstrated that the stromal patch graft could undergo reendothelialization by histological analysis [20]. Similarly, in our study, we noticed that the edematous stroma at the original perforation site grew clear in most cases within a month. The intralamellar patch graft may serve as a scaffold for endothelial cell growth and thus contribute to the favorable endothelial cell density.

In fact, the idea of treating corneal perforation by partial-thickness keratoplasty has become increasingly popular. Research shows that tectonic mini-DSAEK could successfully seal the corneal perforation from within the anterior chamber [21–24]. However, to further achieve better visual acuity, second-stage surgery (mushroom keratoplasty or DALK on DSAEK) is usually required. In our study, the intralamellar patch graft has a similar effect to the mini-DSAEK graft, which could form an internal corneal tamponade and thus achieving better leakproofness and more stable structure. The incidence of pseudo-chamber after traditional DALK in corneal perforation was reportedly 19–41% [8, 9, 25]. Some surgeons also reported recurring perforation after the first DALK; therefore, a second keratoplasty had to be performed [26]. In our study, anatomical success was achieved and no further surgical intervention was required in all patients. Neither graft-host interface leakage nor pseudo-chamber was observed on ASOCT.

Previous research has reported good outcomes of using a thin lamellar graft as the intralamellar patch for sterile corneal perforation [27, 28]. Herein, we performed DALK combined with intralamellar patch for infectious perforation cases for the first time. Considering that incomplete removal of infectious tissue might lead to infection recurrence, we deliberately avoided using the autologous patch graft, except for one case where the patient developed bacterial keratitis secondary to dacryocystitis (No.8). According to the previous research, a high percentage of bacterial keratitis cases can achieve microbiological cure with medical therapy alone [29]. Therefore, after excision of the infectious dacryocyst, we retained a piece of intact stoma layer to serve as the intralamellar patch graft instead of performing the thorough removal of the whole lamella. The patient recovered within a month, and neither infection recurrence nor graft rejection was reported during the follow-up. We preferred to use autologous intralamellar patch graft if condition permitted, for the autologous graft carries no risk of allogeneic rejection. We observed that the graft–host interface haze around the allogeneic patch grafts was significantly more severe than the autologous ones. However, the design and dissection of the recipient bed in the autologous intralamellar patch cases proves to be challenging since there is a demand for sufficient intact stroma tissue within the range of the recipient bed.

The aetiology remains a major determinant of postoperative visual results. Research has found that therapeutic grafts for herpetic keratitis have poor outcomes. In addition to a significant rate of rejection and recurrence, hypoesthesia secondary to the loss of nerve cells may provoke exposure keratopathy [30]. Our study included two patients with herpetic keratitis. Although neither of them developed rejection nor perforation recurrence, the patient with a larger perforation diameter demonstrated poor healing of the surface epithelium. After six months of antiviral therapy and preservative-free artificial tears treatment, the epithelial defect showed no improvement. The patient eventually underwent PK, and the full-thickness graft remained clear. Another aetiology worth attention to is blepharokeratoconjunctivitis (BKC). Corneal perforation secondary to BKC is generally small (≤ 3 mm) and paracentral [31, 32]. Performing PK in such eyes would require larger grafts which carries a higher risk of immune rejection. In our study, performing DALK combined with intralamellar patch graft in patients with blepharokeratoconjunctivitis showed favorable outcomes. All the perforations were paracentral and the interface haze hardly interfered with vision. This technique appears to be an ideal approach for corneal perforation secondary to BKC.

The limitation of this study remains its retrospective nature and the small scale of cases. Since there is plenty of management to prevent a corneal ulcer from developing into a frank corneal perforation, this particular disease is growing rare nowadays. Despite the small sample size, we covered all aspects of common aetiology in this study, which may prove the broad applicability of this innovative surgical approach. The comparison of the interface haze between the autologous and allogeneic intralamellar groups might lack rigor, for the size, location, and aetiology were different among the cases. More research needs to be conducted to further evaluate the vision result between using the allogeneic and autologous intralamellar patch graft.

## Conclusions

DALK combined with intralamellar patch graft was successful in restoring tectonic integrity and improving visual acuity in frank corneal perforation. It could achieve similar leakproofness to that of PK while yielding the advantage of reduced incidence of rejection likes DALK. Furthermore, both the anterior DALK grafts and the allogeneic intralamellar patch grafts could be harvested from the anterior corneal cap of the pre-cut donor cornea prepared for DSAEK to maximize the donor cornea resources. Considering the scarcity of donor corneas in Asian countries, this approach may serve as a secure and effective alternative to PK for frank corneal perforation.

### Electronic supplementary material

Below is the link to the electronic supplementary material.

Supplementary Material 1

Supplementary Material 2

## Data Availability

Data is available from the corresponding author on reasonable request.

## Abbreviations

DALK: Deep anterior lamellar keratoplasty
PK: Penetrating keratoplasty
DSAEK: Descemet-stripping automated endothelial keratoplasty
ASOCT: Anterior segment optical coherence tomography
BCVA: Best corrected visual acuity
IOP: intraocular pressure
PCED: persistent corneal epithelial defects
BKC: blepharokeratoconjunctivitis
