# Supplementary material for: DALK combined intralamellar tectonic patch graft: an alternative approach to treat frank corneal perforation
Source: BMC Ophthalmol. 2023 Oct 27;23:436. doi: 10.1186/s12886-023-03179-7 (PMC10605785; doi:10.1186/s12886-023-03179-7)
Supplement: Supplementary file 2 — Supplementary Material 2 [file 12886_2023_3179_MOESM2_ESM.docx]

**Supplemental Material Legend**

Surgery details

This intraoperative video may give a specific detail about how to harvest an autologous intralamellar patch graft. we peeled off a 10-20 μm layer at the top of the intact deep stroma and flipped it over to cover the perforation site. The procedure of harvesting autologous stroma button demands deft manoeuvres of the surgeon. It takes rich experience to judge the depth of dissection and keep control of the hand movement.
